# Draft Genome Sequence of a Red Basidiomycete Yeast, Symmetrospora coprosmae Strain UCD350, Isolated from Soil in Ireland

**DOI:** 10.1128/MRA.01256-19

**Published:** 2019-10-31

**Authors:** Jameela Almasoud, Laoise McArdle, Ryan Henne, Karen Mathews, Ísla O’Connor, Leah E. McLoughlin, Peadar Ó’Gaora, Kevin P. Byrne, Caoimhe E. O’Brien, Kenneth H. Wolfe, Geraldine Butler

**Affiliations:** a School of Biomedical and Biomolecular Sciences, Conway Institute, University College Dublin, Dublin, Ireland; b School of Medicine, Conway Institute, University College Dublin, Dublin, Ireland; Vanderbilt University

## Abstract

Symmetrospora coprosmae is a red yeast from the subphylum Pucciniomycotina in the phylum Basidiomycota. Here, we present the first genome sequence of *S. coprosmae* strain UCD350, from an isolate collected from soil in Ireland. The genome size is 20.2 Mb.

## ANNOUNCEMENT

*Symmetrospora* species are red ballistosporous yeasts in the subphylum Pucciniomycotina of phylum Basidiomycota (1, 2). Many *Symmetrospora* species were previously called *Sporobolomyces* spp. and were initially placed in the *Erythrobasidium* clade of class Cystobasidiomycetes (3). A more detailed analysis proposed placing Symmetrospora in the family Symmetrosporaceae (1), equivalent to the marina clade described by Wang et al. (2). *Symmetrospora* species form nearly symmetrical ballistoconidia (4). The species studied here has been previously isolated from leaves in New Zealand and Germany under the name *Sporobolomyces coprosmae* (5, 6). The yeast grows as pinkish-red colonies and produces the xanthophyll 2-hydroxytorularhodin (6).

We isolated strain UCD350 from soil in a field in Dublin, Ireland (global positioning system coordinates, 53.336938, −6.270591). The yeast was cultured on yeast extract-peptone-dextrose nutrient agar plates containing chloramphenicol (3% [wt/vol]) and ampicillin (10% [wt/vol]) at 30°C, and the species was identified as *S. coprosmae* by amplifying and Sanger sequencing the internal transcribed spacer of ribosomal DNA (rDNA) using primers ITS1 and ITS4 (7) (GenBank accession number MN540641).

Total genomic DNA was extracted and purified using a QiaAMP DNA minikit (Qiagen). Libraries were generated and sequenced by BGI Tech Solutions (Hong Kong). A total of 1 μg genomic DNA was fragmented using Covaris, purified with an AxyPrep Mag PCR clean up kit, and end repaired, and A tails were added by using an A-tailing mix and incubating at 37°C for 30 min. Illumina adapters were ligated by incubating at 16°C for 16 h. Insert sizes of ∼800 bp were selected, and 150 bases were sequenced from each end with an Illumina HiSeq 4000 instrument, generating 9.5 million spots.

All parameters used for sequence assembly and analysis are available at https://doi.org/10.6084/m9.figshare.9963617.v1. Reads with low quality were trimmed using Skewer v0.2.2 (8). The genome was assembled using SPAdes v3.11.1 (9). Analysis of Illumina sequence data indicates that cross-contamination may result from multiplexing of several samples (10). We used coverage-versus-length (CVL) plots to identify likely contaminants, as described by Douglass et al. (10). Scaffolds with lengths of <1 kb and a coverage of <40× were discarded; coverage of the major nuclear scaffolds was approximately 58×. A contaminating node containing rDNA of Kazachstania servazzii was also removed. Assembly quality was assessed using QUAST v4.6.1 (11). The total assembly size is 20.2 Mb, the *N*_50_ value is 484 kb, the *L*_50_ is 13 scaffolds, and the largest scaffold is 1,583,607 bp. The G+C content in basidiomycetes is normally above 50%, and the G+C content of *S. coprosmae* UCD350 is 59.7%.

**FIG 1:**
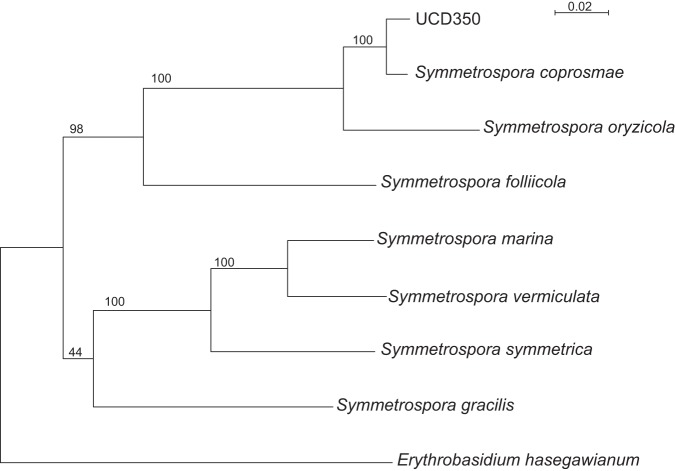
Phylogeny of yeast species in the family Symmetrosporaceae using the combined sequences of SSU rDNA, LSU rDNA D1/D2 regions, ITS regions (including 5.8S rDNA), and *RPB2*, *TEF1*, and *CYTB* genes. Erythrobasidium hasegawianum was used as an outgroup. Alignments were constructed using SeaView (15), and the tree was constructed using maximum likelihood (PhyML, with a general-time-reversible model and 100 replicates). Bootstrap supports are shown. Sequences for all other species were obtained from reference 2.

The mitochondrial genome is a 26,122-bp circular contig with 5,058× coverage (GenBank accession number VUYT01000067). Phylogenetic analyses using seven loci confirmed that UCD350 lies in the family Symmetrosporaceae and is very closely related to the type strain of *S. coprosmae* (Fig. 1).

Variant analysis was carried out using BWA v0.7.12-r1039 (12), SAMtools v0.1.19 (13), and Genome Analysis Toolkit (GATK) v4.0.1.2 using the commands listed at https://figshare.com/articles/Genome_assembly_parameters_for_Symmetrospora_coprosmae_UCD350/9963617 (14). Variants were filtered by removing clusters (5 variants within 20 bp) that were assumed to result from poor read alignment and by applying the following GATK filters: QualByDepth (QD) of <2.0, Mapping Quality (MQ) of <40.0, FisherStrand (FS) of >60.0, StrandOddsRatio (SOR) of >3.0, MappingQualityRankSumTest (MQRankSum) of less than −12.5, and ReadPosRankSumTest (ReadPosRankSum) of less than −8.0. A very small number of potential variants (∼650 heterozygous single nucleotide polymorphisms and ∼90 insertions/deletions) were identified, suggesting that the isolate is haploid.

### Data availability.

This whole-genome shotgun project has been deposited in DDBJ/ENA/GenBank under the accession number VUYT00000000 and the raw reads under SRA accession number SRX6817357. The mitochondrial genome is at GenBank accession number VUYT01000067 and the internal transcribed spacer (ITS) sequence at accession number MN540641. The version described in this paper is the first version. Data are also available under BioProject accession number PRJNA564489.
